# Integrated Circuit Angular Displacement Sensor with On-chip Pinhole Aperture

**DOI:** 10.3390/s20061794

**Published:** 2020-03-24

**Authors:** Udumbara Wijesinghe, Akash Neel Dey, Andrew Marshall, William Krenik, Can Duan, Hal Edwards, Mark Lee

**Affiliations:** 1 Department of Materials Science & Engineering, the University of Texas at Dallas, Richardson, TX 75080, USA; udumbara.wijesinghe@utdallas.edu; 2 Department of Electrical & Computer Engineering, the University of Texas at Dallas, Richardson, TX 75080, USA; akash.dey@utdallas.edu (A.N.D.); andrew.marshall@utdallas.edu (A.M.); 3 Texas Instruments Incorporated, Dallas, TX 75243, USA; w-krenik@ti.com (W.K.); can.duan@ti.com (C.D.); edwards@ti.com (H.E.); 4 Department of Physics, the University of Texas at Dallas, Richardson, TX 75080, USA

**Keywords:** angular sensor, position sensor, displacement sensor, position-sensitive detector, motion tracking

## Abstract

Sensors that remotely track the displacement of a moving object have a wide range of applications from robotic control to motion capture. In this paper, we introduce a simple, small silicon integrated circuit sensor that tracks the angular displacement of an object tagged with a small light source, such as a light-emitting diode (LED). This sensor uses a new angular transduction mechanism, differential diffusion of photoelectrons generated from the light spot cast by the light tag onto a Si anode, that is described by a simple physics model using pinhole optics and carrier diffusion. Because the light spot is formed by a pinhole aperture integrated on the sensor chip, no external focusing optics are needed, reducing system complexity, size, and weight. Prototype sensors based on this model were fabricated and their basic characteristics are presented. These sensors transduce angular displacement of an LED across orthogonal latitudinal and longitudinal arcs into normalized differential photocathode currents with signal linearly proportional to LED angular position across a ± 40° field-of-view. These sensors offer potential performance and ease-of-use benefits compared to existing displacement sensor technologies.

## 1. Introduction

Electronic sensors that can remotely track an object’s displacement in space have numerous applications including feedback control of robotic motion, logging motion of celestial bodies, automated tool alignment, and motion capture [1,2,3,4,5]. Digital imaging systems such as stereo cameras are the most widely known existing technology in this application space [6,7]. These use active pixel sensors (APS) to generate a video, then identify and track an object frame-by-frame using image analysis algorithms [8,9,10]. APS tracking systems are powerful and flexible but require high-quality optical lenses, large area (several cm^2^) megapixel image sensors, and dedicated high-speed image processors. This is often prohibitively expensive or complicated for simple displacement sensing tasks where the object being tracked can be tagged by a small light source, so that forming an image is unnecessary. These simpler tasks often use position-sensitive detectors (PSD) that output a current or voltage giving the 2-d coordinates of a localized light spot cast on a sensor surface. Existing PSDs generally fall into two classes: quadrant photodiodes (QPD) [11,12,13] and lateral effect photosensors (LEP) [13,14,15,16]. Because they do not image, PSD chips are smaller, simpler to fabricate, less expensive, and easier to use than APS chips. However, existing PSDs still require front-end lenses or mirrors to focus light from the light tag onto the sensor chip surface. QPDs are typically used to monitor very small displacement changes at close range, such as the deflection of an atomic force microscope cantilever. LEPs have been used to track a laser-illuminated corner cube reflector at distances > 100 m by monitoring the laser’s retro-reflection [17].

In this article, we introduce a new type of PSD based on an angular transduction mechanism fundamentally different from QPDs and LEPs. In our sensor, the angular displacement of a light tag is transduced to a normalized current signal by differential diffusion of photoelectrons generated from a localized light spot cast by a light tag through a pinhole aperture. Because of a resemblance to an ancient star-tracking navigational tool called an astrolabe [18], we call this sensor an integrated circuit (IC) astrolabe. A simple physical model of the angular transduction is developed, and we show this model can be realized in a prototype fabricated using standard Si integrated circuit processing. A single IC astrolabe chip can track angular displacement across two orthogonal arcs; two or more such sensors on a fixed baseline geometry can fix 3-d spatial position via triangulation. 

An IC astrolabe PSD offers several significant potential advantages over existing displacement sensing technologies. The IC astrolabe uses an on-chip integrated pinhole aperture to focus light from the light tag onto the sensor surface [19], eliminating the need for front-end optical components. This reduces system size, weight, and cost compared to all existing APS and PSD based displacement sensing systems. Since no lenses or mirrors are needed to be kept in alignment, IC astrolabe systems should have greater mechanical and thermal stability. Unlike QPDs, IC astrolabes do not require the light spot to overlap multiple photocathodes, making the IC astrolabe’s response insensitive to light spot shape and permitting it to follow angular displacement changes over a wider field-of-view. Unlike LEPs, astrolabes do not use the lateral photoelectric effect that requires a p-n junction covering the entire sensor surface area. The IC astrolabe uses p-n junctions with interface area significantly smaller than in an LEP of same sensor surface area, reducing dark current and parasitic capacitance to potentially improve sensitivity and speed. LEPs are also strongly affected by surface recombination and the material quality of a thin heavily doped layer [20], problems avoided in the IC astrolabe.

## 2. IC Astrolabe Concept and Angular Transduction Model

**Figure 1:**
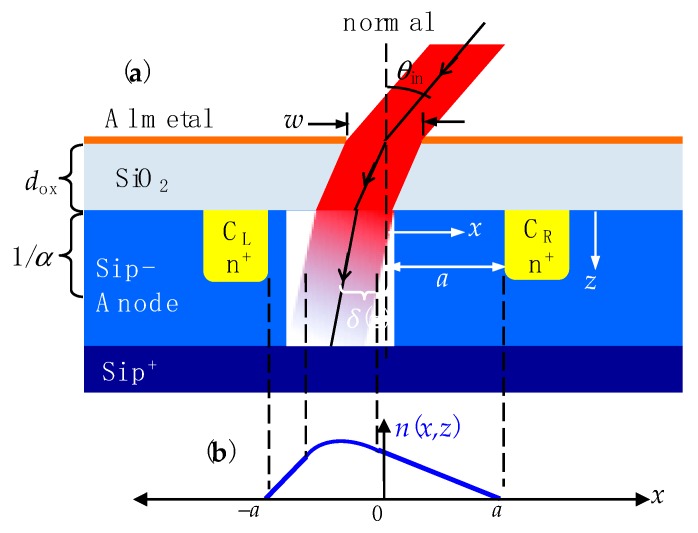
(**a**) Illustration (not to scale) of the angular displacement sensor cross-section. (**b**) The functional form of photoelectron density *n*(*x*,*z*) as a function of *x* at a fixed *z*.

Figure 1a illustrates (not to scale) the design concept of an IC astrolabe sensor device, drawn in cross-section through a Si chip. The sensor consists of a heavily p^+^ doped layer as ground contact, a moderately p-doped layer as an anode, a SiO_2_ layer of thickness *d*_ox_ as a refracting spacer layer, and aluminum metallization at the surface. A pinhole aperture of diameter *w* is opened in the Al metal. Two identical n^+^-doped cathodes, C_L_ (left) and C_R_ (right), are implanted in the anode at distance ±*a* from the centerline. With a point light source tag far from the aperture (i.e., at distance >> *w*), the light tag can be taken to be at infinity so parallel light rays (drawn as a red beam in Figure 1a) are incident at angle *θ*_in_ relative to the normal. Because a pinhole aperture has infinite depth-of-field [19], no focusing optics are needed. Light rays refract through the SiO_2_ and the Si anode with net refraction angle *θ*_refr_ = sin^−1^[(sin*θ*_in_)/*n*_Si_] where the real index *n*_Si_ ≥ 3.5 for above-bandgap light [21]. Because *n*_Si_ is large, we can approximate *θ*_refr_ ≈ *θ*_in_/*n*_Si_. At a depth *z* into the anode, the center of the light beam is displaced a horizontal distance *δ*(*z*) = (*d*_ox_ + *z*)*θ*_in_/*n*_Si_ in the *x*-direction from the centerline.

The light is locally absorbed in the anode and generates photocarriers in the anode. With cathodes positively biased at the same potential relative to the anode (taken as ground), photoelectrons will diffuse to and be collected by the cathodes. If *θ*_in_ = 0, the situation is symmetric among the cathodes so in Figure 1a the left cathode current *I*_L_ = right cathode current *I*_R_. If *θ*_in_ ≠ 0 then the light spot will be closer to one cathode (C_L_ in Figure 1a) than the other (C_R_). More photoelectrons will then diffuse to C_L_ than to C_R_, resulting in *I*_L_ > *I*_R_. Normalizing the difference photocurrent (*I*_L_ − *I*_R_) to the total (*I*_L_ + *I*_R_) eliminates extensive effects like cathode area and incident light intensity, so *θ*_in_ is transduced to a normalized differential photocurrent (*I*_L_ − *I*_R_)/(*I*_L_ + *I*_R_).

This intuitive device physics can be quantified in an analytical model by calculating the diffusion of photoelectrons in the anode. We make the simplifying assumption that every photon incident on the anode creates an electron–hole pair and neglect recombination loss. Taking the light intensity in the anode to be exponentially decaying with depth, characterized by a wavelength-dependent absorption coefficient *α* [21], charge conservation gives the total current as:

(1) $$I_{L}+I_{R}=-qw\int_{0}^{\infty }U_{0}\exp\left(-\alpha z\right)\;dz$$

where *q* is the electron charge and *U*_0_ is the photon flux at the anode surface. The upper integration limit can be taken to ∞ when the anode thickness is > 1/*α* with little loss of accuracy. 

Photoelectrons generated in the illuminated region diffuse in the ±*x*-direction towards the cathodes. The photoelectron density *n*(*x*,*z*) at any given depth *z* is obtained from the time-independent 1D diffusion equation [22]. Inside the illuminated region where photoelectrons are generated:

(2a) $$D\frac{\partial^{2}n(x,z)}{\partial x^{2}}+U_{0}\exp\left(-\alpha z\right)=0$$

where *D* is the diffusion coefficient and *U*_0_exp(–*αz*) is the generation term. Equation (2a) is solved by a quadratic function of *x*. Outside the illuminated region, where no photocarriers are generated:

(2b) $$D\frac{\partial^{2}n(x,z)}{\partial x^{2}}=0$$

which is solved by a linear function of *x*. The solutions *n*(*x*,*z*) to Equations (2a) and (2b) must be continuous at the boundaries between illuminated and un-illuminated regions. Also, taking each cathode to be a perfect recombination center of photoelectrons diffusing in from the anode, we require *n*(±*a*,*z*) = 0. These boundary conditions determine the arbitrary constants in the solutions of Equations (2a) and (2b). The functional form of *n*(*x*,*z*) is sketched in Figure 1b. 

The photocurrent can be obtained by applying Fick’s law [23] relating diffusion to current and integrating over depth using Equation (1), resulting in a normalized photocurrent signal *Σ* given by:

(3) $$\Sigma =\frac{I_{L}-I_{R}}{I_{L}+I_{R}}=\frac{1}{n_{\mathrm{Si}}a}\left[d_{\mathrm{ox}}+\frac{1}{\alpha }\right]\theta_{\mathrm{in}}$$

where *d*_ox_ is the SiO_2_ layer thickness and *a* is the center-to-cathode distance as illustrated in Figure 1a. 

As long as the approximation *θ*_refr_ ≈ *θ*_in_/*n*_Si_ holds Equation (3) shows that *Σ* is linearly proportional to *θ*_in_, with a proportionality coefficient that depends on detector layout geometry (through *a* and *d*_ox_) and light wavelength (through *n*_Si_ and *α*). This linearity can be expected to break down when *θ*_ιn_ is large enough that the approximation *θ*_refr_ ≈ *θ*_in_/*n*_Si_ is no longer valid or when refracted light shines directly on a cathode. Photoelectrons generated in a cathode do not need to diffuse to be collected and so will lead to signal saturation. From Figure 1a, as *θ*_in_ increases light will initially intersect a cathode below the anode surface where the intensity is weaker, so there should be a gradual approach to saturation. Thus, we expect the onset of a sub-linear *Σ* vs. *θ*_in_ response at relatively high *θ*_in_.

**Figure 2:**
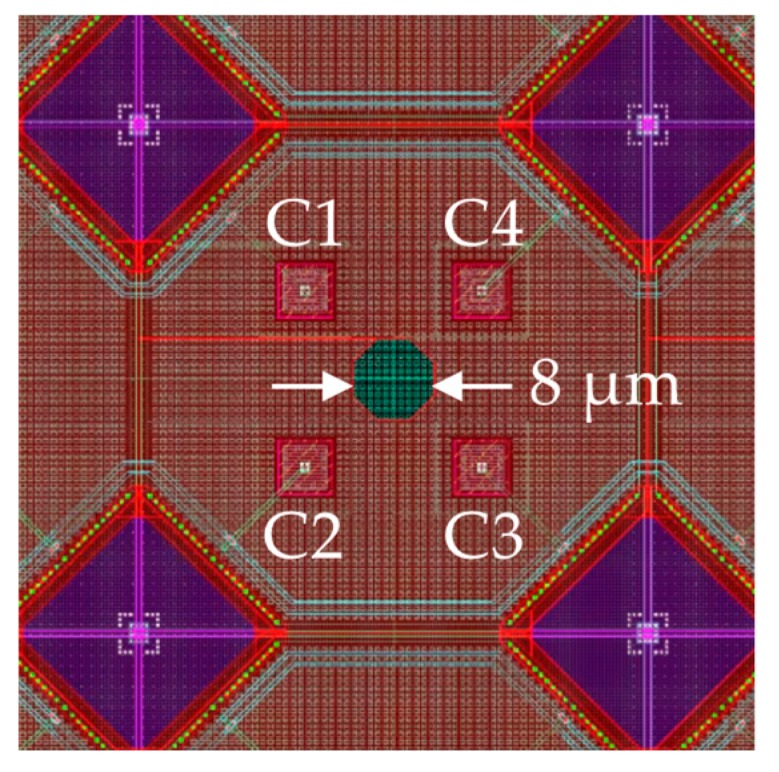
Plan view design drawing of an integrated circuit (IC) astrolabe unit cell.

We note that Figure 2 would be equivalent to a QPD if the spacing between cathodes 2*a* → 0 so that they bordered each other. In that case, *Σ* depends only on the fraction of incident light that shines directly onto C_L_ vs. C_R_. As in all QPDs, *Σ* would then saturate when *θ*_in_ is such that the light spot illuminates only C_L_. It is then clear that separating the cathodes, using photoelectron diffusion to carry the signal to each cathode, increases the angular detection range. Using fixed dimensions *w* and *d*_ox_ from Figure 2, a straightforward geometrical calculation gives the field-of-view for a QPD cathode configuration (2*a* → 0) to be approximately half of that reported in Section 4.2 for a cathode layout with 2*a* = 12 µm.

## 3. Prototype IC Astrolabe Layout and Fabrication

We tested the angular transduction model of by fabricating prototype IC astrolabe sensors using a 0.18 µm generation Si complementary-metal-oxide-semiconductor process line. The cross-sectional structure follows Figure 1a. The anode consisted of a 20 µm thick p-doped layer (10^15^ to 10^17^ cm^–3^) above a deep p^+^ layer (> 10^18^ cm^–3^). Photocathodes were formed in the anode by ion implanting n^+^ wells (~10^17^ cm^–3^) to a depth of 10 µm into the anode. A *d*_ox_ = 10 µm layer of SiO_2_ was grown on top of the anode to support an aluminum metal box. Figure 2 shows a plan view of the design drawing (to scale) of an IC astrolabe unit cell. The reddish shading represents the top Al layer. An 8 µm diameter pinhole aperture in the Al looks down onto the anode surface, shaded green. Four photocathodes underneath the Al on the anode surface are shown as small squares labeled C1, C2, C3, and C4, at the corners of a 12 × 12 µm square centered below the aperture. The purple diamonds at the corners are contacts for the cathodes’ electrical leads. An IC astrolabe chip consisted of an 8 × 8 array of such unit cells, each cell 50 × 50 μm^2^, for a total sensor area of 0.4 mm × 0.4 mm. All 64 C1 cathodes were connected in parallel to a common bonding pad to output current *I*_1_, and similarly for C2, C3, C4, and their currents *I*_2_, *I*_3_, *I*_4_. Each chip had a common anode bonding pad used as the circuit ground.

This IC astrolabe tracks angular displacement in two orthogonal arcs. Sweeping a light tag along a latitudinal arc (left-to-right in Figure 2, like latitude lines on a map), the total left-side photocurrent is *I*_L_ = *I*_1_ + *I*_2_, and the right-side current is *I*_R_ = *I*_3_ + *I*_4_. Thus, the normalized latitudinal signal *Σ*_LAT_ is:

(4a) $$\Sigma_{\mathrm{LAT}}=\frac{\left(I_{1}+I_{2}\right)-\left(I_{3}+I_{4}\right)}{I_{1}+I_{2}+I_{3}+I_{4}}$$

Similarly, sweeping a light tag along a longitudinal arc (top-to-bottom in Figure 2, like longitude lines on a map) the total top-side photocurrent is *I*_1_ + *I*_4_, and the bottom-side current is *I*_2_ + *I*_3_. The normalized longitudinal signal *Σ*_LON_ is then:

(4b) $$\Sigma_{\mathrm{LON}}=\frac{\left(I_{1}+I_{4}\right)-\left(I_{2}+I_{3}\right)}{I_{1}+I_{2}+I_{3}+I_{4}}$$

*Σ*_LAT_ and *Σ*_LON_ are orthogonal. For general 2-d angular displacements, the azimuthal angle *ϕ* relative to the latitudinal direction is given by *ϕ* = tan^−1^[*Σ*_LON_/*Σ*_LAT_].

## 4. Basic Performance Characteristics of IC Astrolabe Prototype

### 4.1. Measurement Methods

Two IC astrolabe chips of identical design were tested and showed quantitatively consistent angular transduction characteristics. Each was mounted in an uncovered 8-pin ceramic dual inline package (DIP) and Au wire bonds were made from the four cathodes and one anode (ground) contact pads on the chip to the pin leads on the DIP. To measure performance, a DIP was plugged into a vector board socket and mounted on a Thorlabs PRMTZ8 digitally controlled rotation stage, with the chip centered on the rotation axis. A light-emitting diode (LED), fixed in position, was used as a light tag. The data shown in Section 4.2 and Section 4.3 were taken with a 660 nm (red) LED light tag. LED wavelengths of 830 nm (infrared) and 525 nm (green) were also used, as discussed in Section 4.4. The LED was placed at various distances from 0.2 to 1.0 m from the sensor, facing normal to the plane of the vector board when the rotation stage was set at 0°. We found the only effect of increasing distance was a decrease in illumination power incident on the detector, which could be compensated for by increasing LED brightness. Consequently, we characterized angular transduction performance at constant illumination power, rather than distance. The illumination power incident onto the DIP package was determined by placing a Thorlabs S130C power meter directly in front of the sensor chip before rotation measurements. For the data shown in Section 4.2, at any distance or wavelength, LED intensity was adjusted so that the measured incident power was 300 ± 10 µW over the 9.5 mm diameter aperture of the power meter. Assuming uniform illumination of the power meter area, the power incident on the 0.4 mm × 0.4 mm sensor area was then 0.68 µW. 

During measurements, all anode–cathode pn junctions were reverse biased at a constant 1.5 V. At this bias, the photocathodes were current sources, so a quad transimpedance amplifier (TIA) (LMP2234) was mounted on the same vector board and connected to the astrolabe DIP via short (~1 cm) soldered wires. The TIA generated four output voltages, *V_n_* = −*RI_n_* (*n* = 1 to 4), directly proportional to the cathode currents *I_n_*, where *R* = 60 MΩ was the fixed transimpedance.

Angular sensing measurements were performed by rotating the DIP from –90° to +90° relative to the LED in 1° steps, where 0° is normal incidence. At each angle, the four photocurrent signals *V*_1_, *V*_2_, *V*_3_, and *V*_4_ from the TIA were measured using Keithley 2401 source measure units operating in voltmeter mode with an integration time of 217 ms. In all cases, photocurrent signals were first measured with LED off in both dark (black box) and ambient laboratory light conditions. The Thorlabs S130C power meter read about 30 nW in the dark and 30 µW in ambient light with the LED off. Photocurrents were then measured with LED on against both dark and ambient background light conditions. All normalized signal data *Σ*_LAT_ and *Σ*_LON_ are calculated using the difference between LED on and LED off in ambient light conditions, i.e., ∆*V_n_* = [*V_n_*(LED on) – *V_n_*(ambient light)] for each cathode *n* at each rotation angle.

**Figure 3:**
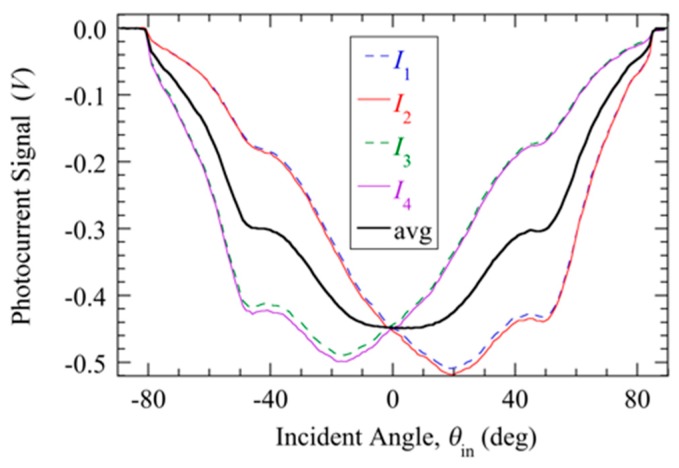
Photocurrent signal from transimpedance amplifier for each cathode current.

Figure 3 shows the photocurrent signal ∆*V_n_* as defined in the preceding paragraph for each cathode as a function of *θ*_in_ using a 660 nm LED light tag swept latitudinally from –90° < *θ*_in_ < 90°. Each current is angle-sensitive with a horizon of nearly ± 80°, beyond which insufficient light from the LED enters the pinhole. Also shown is the average of the four cathode signals, which has a maximum signal corresponding to 7.5 nA at *θ*_in_ = 0°.

### 4.2. Horizon, Field-of-View, and Angular Sensitivity

**Figure 4:**
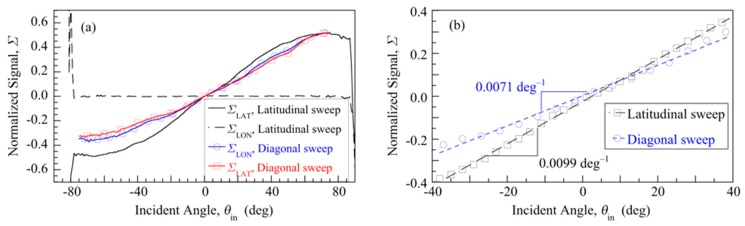
(**a**) Normalized signals for a latitudinal sweep (black solid and dashed lines) and a diagonal sweep (red and blue symbols and lines). (**b**) Details of the -40° to 40° portion of (a). Dashed lines are linear fits to the data, with linear slopes indicated.

Figure 4a shows *Σ*_LAT_ and *Σ*_LON_ for: a i) latitudinal arc sweep (black solid and dashed curves), and ii) diagonal arc sweep (red and blue symbols and curves) at azimuth angle *ϕ* ≈ 45°, i.e., in the direction from cathodes C2 to C4 in Figure 2. For the latitudinal sweep, *Σ*_LAT_ is linear with *θ*_in_ for |*θ*_in_| < 40°, consistent with Equation (3). At higher *θ*_in_, *Σ*_LAT_ becomes sub-linear, as expected physically from the discussion following Equation (3). The physical horizon at which light incident on the aperture no longer hits the anode is near ± 80°, close to the geometrical limit of ± 90°. However, although the signal-to-noise ratio is still good for 50° < |*θ*_in_| < 80°, *Σ*_LAT_ becomes too weakly dependent on *θ*_in_ to determine the angle for |*θ*_in_| > 50°. We, therefore, define the linear field-of-view to be the *θ*_in_ where |*Σ*_LAT_| falls 1 dB below its low angle linear extrapolation, in analogy to gain compression of an amplifier. This linear field-of-view is around ± 40°. Meanwhile, the latitudinal sweep *Σ*_LON_ is insensitive to *θ*_in_ and essentially zero, demonstrating the orthogonality of *Σ*_LON_ and *Σ*_LAT_. For the diagonal sweep, *Σ*_LON_/*Σ*_LAT_ ≈ 1 as expected since tan(*ϕ*) = 1.

The angular sensitivity of this kind of sensor measures how large the normalized signal change is when the angular displacement of the light tag changes by 1° within the sensor’s linear field-of-view. Figure 4b expands the plot of Figure 4a to emphasize the low angle regime |*θ*_in_| < 40°, where least-squares linear fits are made to *Σ*_LAT_ vs. *θ*_in_ for both a latitudinal and a diagonal arc sweep. From the data of Figure 4b, we define the angular sensitivity *S* = ∆*Σ*/∆*θ*_in_ as the slope of these linear fits to *Σ* vs. *θ*_in_ data for |*θ*_in_| < 40°. For the latitudinal sweep, *S*_LAT_ = 0.0099 deg^−1^, whereas for the diagonal sweep *S*_DIA_ = 0.0071 deg^−1^. The ratio *S*_DIA_/*S*_LAT_ is close to 2^–1/2^ = cos(*ϕ*), as expected. *S* was measured at several incident illumination powers between 0.01 and 1.5 µW and found to be essentially independent of power. 

### 4.3. Basic Noise Characteristics

**Figure 5:**
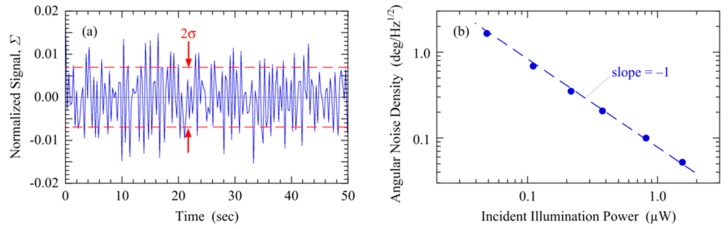
(**a**) Example time record of the normalized signal at *θ*_in_ = 0°. (**b**) Log–log plot of angular noise density vs. incident illumination power from 660 nm light tag. Dashed line shows a slope of -1.

The basic noise characteristics of this IC astrolabe prototype were evaluated. Time records of the normalized signal were recorded for 50 s using an integration time of *τ* = 21.7 ms at several incident powers with the sensor and LED light tag enclosed inside a black box to isolate the system from background ambient light noise. Figure 5a shows an example time record of the normalized signal *Σ* at *θ*_in_ = 0° using a 660 nm LED and 0.38 µW of illumination power incident on the sensor area. From this data, the total standard deviation of the fluctuations about the mean is 2*σ* = 0.0139, so the signal noise density is 2*στ*^1/2^ = 2.05 × 10^–3^/Hz^1/2^. Dividing by the angular sensitivity *S*_LAT_= 0.0099 deg^−1^ for a latitudinal sweep gives the angular noise density *η* = 2*στ*^1/2^/*S*_LAT_ = 0.207 deg/Hz^1/2^. *η* measures how small an angular change can be reliably measured using a given illumination power and bandwidth.

Several such time records were recorded at different LED incident illumination powers. Figure 5b plots the dependence of *η* on illumination power using a 660 nm LED light tag. On a log–log plot, the data fall almost exactly on a line with slope = -1, showing that *η* varies inversely with illumination power. Since *S* was found to be independent of incident LED power, this dependence of *η* on power is entirely due to an increase in signal noise density 2*στ*^1/2^ with decreasing illumination power. The inverse dependence of noise on LED power suggests that the noise is dominated by the detector or the light tag, rather than by the TIA or voltmeter. Possible mechanisms of noise generation, including shot, interface roughness and traps, generation–recombination noise, and interference are being investigated.

### 4.4. Light Tag Wavelength Dependence

In addition to the 660 nm LED light tag, angular sensitivity and noise were measured with 830 nm (above bandgap infrared) and 525 nm (green) wavelength LEDs. Table 1 summarizes *S*_LAT_ and *η* for the three LED wavelengths. At all wavelengths, *η* in Table 1 was determined using the same incident illumination power while *S*_LAT_ was found to be independent of illumination power. 

Table 1 also shows that *η* is lowest for 660 nm compared to 830 nm and 525 nm. The signal noise density (in Hz^–1/2^) using 525 nm is roughly 20% higher than using either 660 or 830 nm. The elevated noise density at 525 nm may arise because the penetration depth 1/*α* in Si is shallowest at 525 nm among the three wavelengths tested. As a consequence, 525 nm light generates photocarriers closest to the Si/SiO_2_ interface where interface roughness and dangling bonds may act as significant noise sources. The signal noise density (in Hz^–1/2^) using 830 nm is comparable to that using 660 nm. The higher angular noise density (in deg/Hz^1/2^) at 830 nm is primarily due to its lower *S*_LAT_ value.

*S*_LAT_ is distinctly smaller at 830 nm compared to 660 nm and is nearly the same for 660 and 525 nm. This observed behavior is the opposite of what is predicted by Equation (3). Using the wavelength-dependent values of *n*_Si_ and 1/*α* in Reference [21], Equation (3) predicts a monotonically decreasing sensitivity *dΣ/dθ*_in_ with decreasing wavelength. A possible reason for the discrepancy is the neglect of anode recombination in the model leading to Equation (3). The 830 nm light can generate photoelectrons well below the depth of the cathode, requiring those photoelectrons to diffuse upward via a longer path that yields a higher probability of recombination and hence loss of signal.

### 4.5. Potential Future Improvements to the Prototype IC Astrolabe

The IC astrolabe sensors presented here were intended to prove the viability of the angular transduction mechanism described in Section 2. The performance characteristics shown for the first realization of this sensor can be improved using the existing performance data for guidance. For example, signal noise density (Figure 5a) is likely limited by the small *w* = 8 µm pinhole aperture restricting the amount of light reaching the anode. Thus to decrease signal noise, a larger *w* could be used. However, a larger aperture has the possible disadvantage of also decreasing the angular sensitivity *S* (Figure 4b) because the larger light spot on the anode is not as spatially localized (e.g., in the limit *w* → ∞ the sensor is uniformly illuminated at any *θ*_in_, so *S* → 0.) As a consequence, there should be an aperture size that minimizes the angular noise density *η*, and it is of interest for future work to determine whether such an optimum aperture exists and what it might be. Alternatively, angular noise density could be improved significantly by using an array of 50 µm diameter microlenses, one on top of each unit cell pinhole, to gather more light to the aperture. Using a microlens array has the advantage of increasing the amount of light illuminating the anode while preserving the localization of a small light spot with a small aperture. A microlens array has the disadvantage of adding complications and hence cost to the sensor fabrication.

## 5. Summary

In summary, we introduced a new electronic angular displacement sensor, the IC astrolabe, where the angular transduction mechanism is based on pinhole optics and 1-d photoelectron differential diffusion. An integrated pinhole aperture precludes the need for external optics, making the IC astrolabe much simpler to use compared to all existing positions or angular displacement detectors. The differential diffusion mechanism allows IC astrolabes to have a much wider field-of-view compared to quadrant photodiodes and potentially much lower parasitic capacitance and dark current compared to lateral effect photosensors. Since they do not image, IC astrolabes can be more cost-effective compared to active pixel sensor-based motion tracking. Prototype realizations of this model were fabricated using standard Si integrated circuit technology. These prototypes can track a light tag over a ±40° field-of-view with a linear response to angular changes in two orthogonal arcs. The basic performance characteristics of these prototype sensors were measured and found to be qualitatively consistent with the expectations of the device model.

## Figures and Tables

**Table 1:** Sensitivity and angular noise density at different light tag wavelengths.

LED Wavelength (nm)	*S*_LAT_ (deg^−1^)	*ηP*_in_ (deg·µW/Hz^1/2^)
525	0.0094	0.257
660	0.0099	0.207
830	0.0069	0.263
